# Enhanced expression of IL-34 in an inflammatory cyst of the submandibular gland: a case report

**DOI:** 10.1186/s41232-018-0069-6

**Published:** 2018-07-10

**Authors:** Muhammad Baghdadi, Kozo Ishikawa, Hiraku Endo, Yui Umeyama, Tsukasa Ataka, Haruka Wada, Yumiko Oyamada, Naoki Hyakushima, Ken-ichiro Seino

**Affiliations:** 1 Division of Immunobiology, Institute for Genetic Medicine, Hokkaido University, Kita-Ku, Kita-15 Nishi-7, Sapporo, 060-0815 Japan; 2 Tonan Hospital, Kita-Ku, Kita-4 Nishi-7, Sapporo, 060-0004 Japan

**Keywords:** Inflammatory cyst, Submandibular gland, Inflammation, Interleukin 34, Ductal epithelium, Endothelium, Immune infiltration

## Abstract

**Background:**

Cysts of the salivary glands are common lesions that occur in the context of various etiologies. Although the diagnostic importance of cysts in salivary gland diseases has been well studied, molecular mechanisms that control the related pathological process remain largely unknown. IL-34 is a novel cytokine that was discovered recently as a tissue-specific ligand of colony stimulating factor-1 receptor. Since its discovery, accumulating evidence has revealed emerging roles of IL-34 in various pathological conditions and has been suggested to correlate remarkably with inflammation. In this study, we report a medical case of an inflammatory cyst within the submandibular gland, through which evaluating the possible involvement of IL-34 in salivary gland disorders.

**Case presentation:**

A 37-year-old male patient suffered from a sudden swelling in the right submandibular region, started initially small and had gradually increased in size to reach 3–4 cm in 1 week, accompanied by pain and local fever. Ultrasonography and MRI imaging revealed the existence of a well-defined cystic lesion with sharp borders measuring 39.8 mm × 19.7 mm within the right submandibular gland. The cyst was removed surgically, and the diagnostic decision was determined based on histopathological observations as an inflammatory cyst in the submandibular gland. Sections were generated from different regions of the surgically resected inflammatory cyst and used to examine IL-34 expression by immunohistochemistry compared to normal salivary gland tissues. Immunohistochemical staining showed enhanced expression of IL-34 in the ductal epithelial cells and endothelial cells of blood vessels, with a tendency to be accompanied with high infiltration of immune cells, which suggests a possible involvement of IL-34 in the pathogenesis of salivary gland inflammation.

**Conclusions:**

In this report, we introduce interesting findings of enhanced IL-34 expression in a case of an inflamed submandibular gland. Our findings emphasize the pathological roles of IL-34 as an inflammation amplifier and angiogenic enhancer in inflammatory conditions, such as in salivary gland disorders.

**Electronic supplementary material:**

The online version of this article (10.1186/s41232-018-0069-6) contains supplementary material, which is available to authorized users.

## Background

Several pathological conditions affect the salivary glands, and the causes range from infection (bacterial or viral) to autoimmune and neoplastic etiologies [1]. Enlargement of the salivary glands is also frequently seen in inflammatory disorders of unknown etiology [1]. During the inflammatory process, the affected gland is characterized by swelling, pain, tenderness, and fever [1]. Additionally, cysts occur frequently in the salivary glands during the acute phase of inflammation. Although several studies have focused on the diagnostic importance of inflammatory cysts in salivary glands, these studies still lack a molecular insight into factors that relate to inflammation and affect or mediate the pathological process of salivary glands disorders.

IL-34 is a novel cytokine that was reported for the first time in 2008 as an alternative ligand of CSF-1R that controls myeloid cell survival, proliferation, and differentiation [2]. IL-34 shows tissue-specific expression under physiological conditions, in the skin by keratinocytes and in the brain by neurons, where it mediates the development and maintenance of Langerhans cells and microglia, respectively [3–5]. Under pathological conditions, IL-34 is produced by a wide range of cells and plays important roles in the etiology of various diseases including infection, inflammation, autoimmunity, metabolic disorders, and cancer [6]. Experimentally, accumulating evidence indicates that IL-34 expression is induced in stressed cells under inflammatory conditions and, consequently, affects the cellular and molecular signaling networks that determine the pathological outcomes in various diseases [6]. Indeed, IL-34 has been suggested to serve as a novel biomarker to monitor disease progression and severity, in addition to prognostic importance such as in cancer [6–9].

From these backgrounds, we expected a potential role of IL-34 in the inflammatory disorders of salivary glands. Here, we report for the first time a case of an inflammatory cyst in the submandibular gland that showed enhanced expression of IL-34 in the endothelium and ductal epithelium which is accompanied with immune infiltration, indicating a possible involvement of IL-34 in the etiology of these disorders.

## Case presentation

**Fig. 1:**
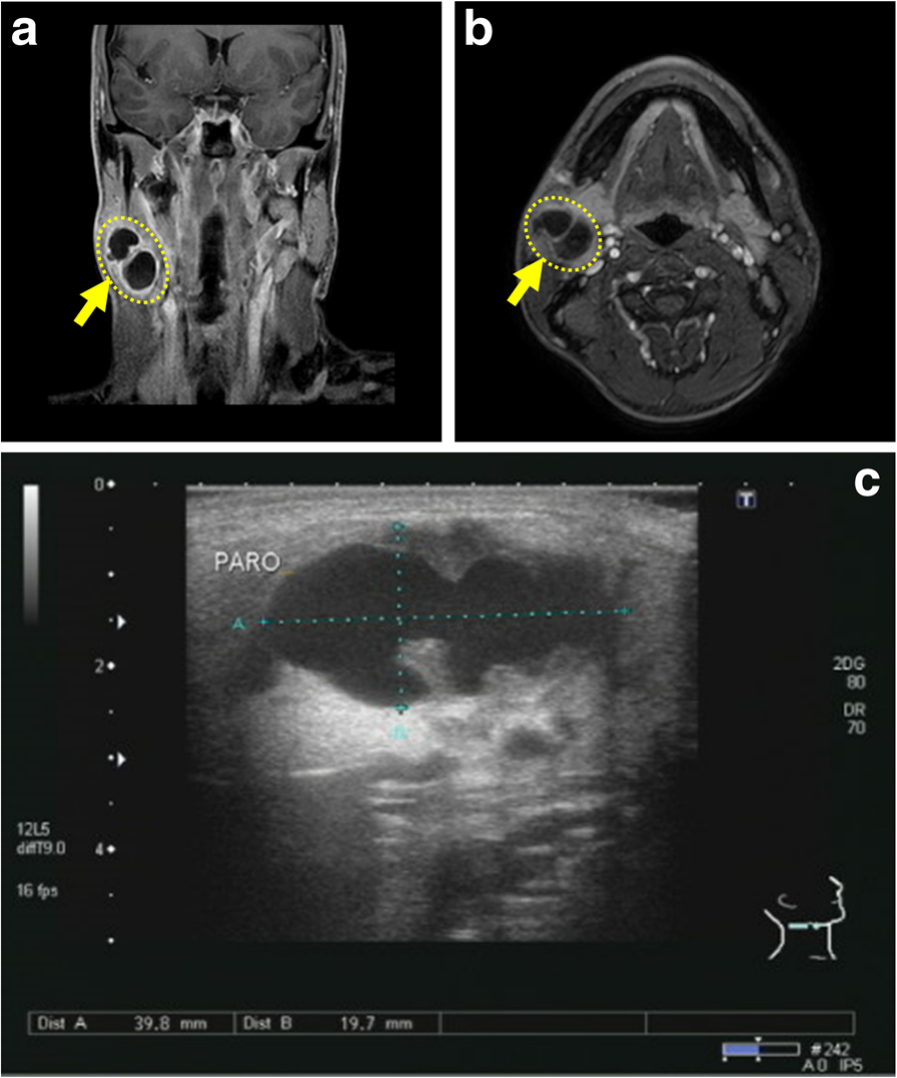
An inflammatory cyst within the submandibular gland. Coronal (**a**) or axial (**b**) *T*_2_-weighted MRI images of the head and neck demonstrating the existence of a cyst within the right submandibular gland (yellow arrows). Ultrasonic imaging of the inflamed submandibular gland (**c**) showed the inflammatory cyst as a black hole with sharp borders, measured 39.8 mm × 19.7 mm in diameter

A 37-year-old male patient suffered from a sudden swelling in the submandibular region of the right side of the neck. The swelling started initially small and had gradually increased in size to reach 3–4 cm in 1 week, accompanied by pain and local fever. The patient’s history was unremarkable. On extraoral examination, a single, localized, well-defined, ovoid swelling was present in the right submandibular region. On palpation, the swelling was tender, mobile, soft in consistency, compressible but not reducible. Intraoral examination showed no abnormalities. The patient was treated with an antibiotic regimen for 10 days without any significant improvement. All serological parameters were within normal limits, and testing of HBs-Ag, HCV-Ab and HIV-Ag/Ab was negative. Ultrasonography and MRI imaging of the head and neck revealed a well-defined cystic lesion with sharp regular borders measuring 39.8 mm × 19.7 mm within the right submandibular gland (Fig. 1). The cyst was then enucleated under general anesthesia. Diagnosis decision was determined on histopathological observations as an inflammatory cyst in the submandibular gland. The patient was reviewed after 6 months with no evidence of recurrence as confirmed by ultrasound imaging.

**Fig. 2:**
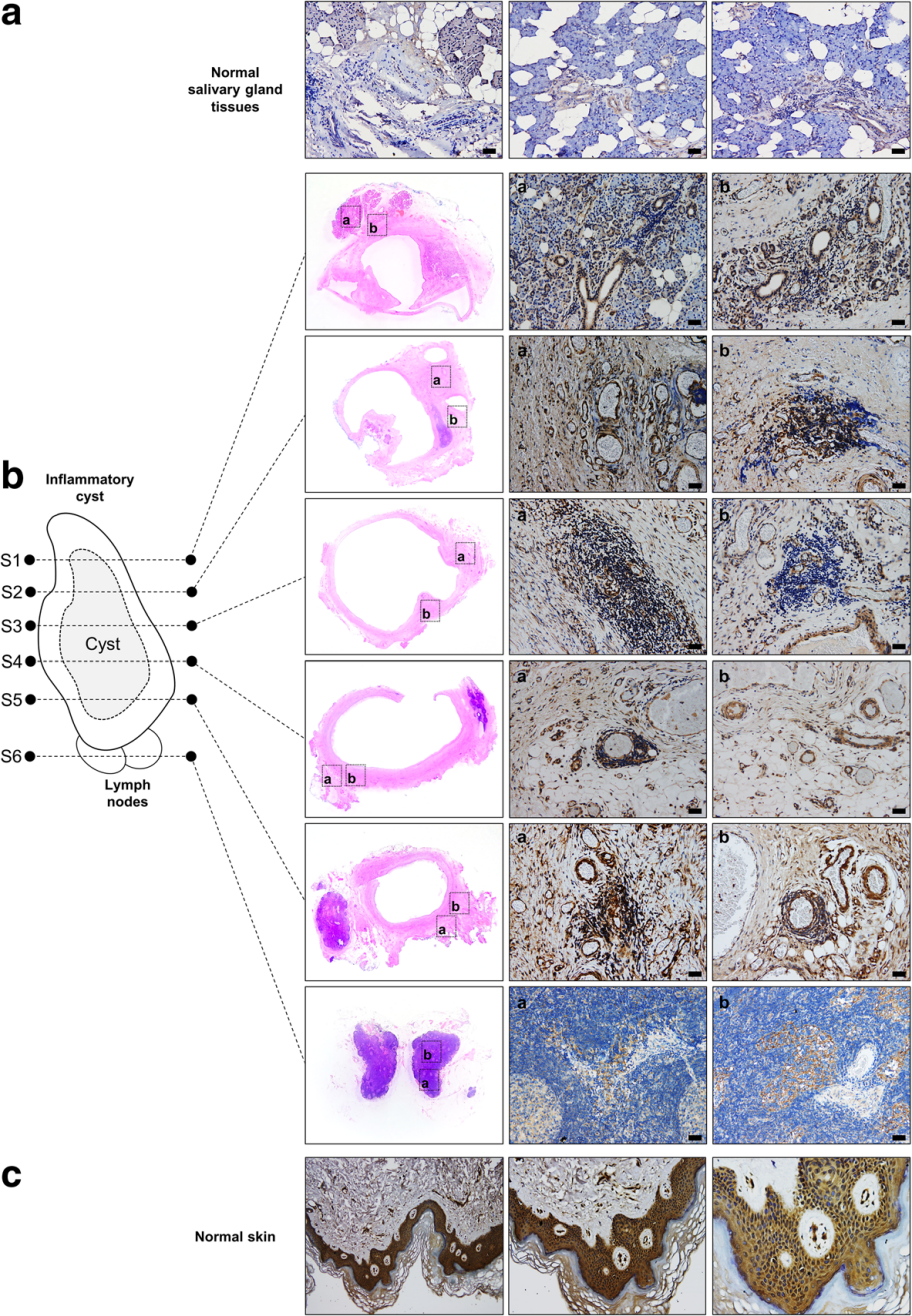
Enhanced IL-34 expression in an inflammatory cyst of the submandibular gland compared to normal salivary gland tissues. Representative data of immunohistochemistry staining of IL-34 in normal salivary gland tissues (**a**) or inflamed submandibular gland (**b**). IL-34 staining was performed on sections obtained from five different regions of the surgically resected inflammatory cyst (from S1 to S5) in addition to one sample from the adjacent swollen lymph nodes (S6). H & E staining of sections is shown in the left column, followed by two representative data of IL-34 staining in tissues surrounding the inflammatory cyst. Squares within H & E photography indicate the correspondent positions of IHC data. Scale bars, 20 μM. The specific staining of IL-34 in keratinocytes of normal skin is shown in **c** at different magnifications as a positive control to confirm the specificity of the anti-IL-34 antibody

To examine the possible involvement of IL-34 in the inflammatory response observed in this case, 5-μm thickness sections were prepared from FFBE tissue samples obtained from five different regions of the surgically resected inflammatory cyst in addition to one sample from the adjacent swollen lymph nodes (Fig. 2). The expression of IL-34 was examined in these sections as compared to normal human salivary gland tissues (OriGene, Catalog No: CS811918, Case ID: CU0000012638, Sample ID: PA0000964A). Immunohistochemistry staining was performed using a specific antibody against IL-34 (EMD Millipore, Catalog No: MABT493, 1:200 dilution, 4 °C overnight) followed by secondary antibody reaction (ImmPRESS UNIVERSAL REAGENT, Anti-Mouse/Rabbit IgG PEROXIDASE Vector, Catalog No: MP-7500, 30 min at room temperature). Peroxidase/DAB was added, and the sections were counterstained with hematoxylin.

By microscopic observation, normal salivary gland tissues showed typical histology, composing of serous acini mixed with adipocytes and ducts lined by cuboidal epithelial cells (Fig. 2a). IL-34 expression was undetectable in serous acini or endothelium, but can be observed at very weak levels in the epithelium of the ductal system (Fig. 2a). As expected, IL-34 showed enhanced expression levels in tissues of the submandibular gland surrounding the inflammatory cyst (Fig. 2b). In particular, IL-34 showed remarkable enhancement in the cuboidal epithelial cells of the ductal system and interestingly in endothelial cells lining blood vessels within theaffected gland (Fig. 2b). IL-34 showed very weak staining in some areas within the adjacent swollen lymph nodes (Fig. 2b). The specificity of the anti-IL-34 antibody was confirmed by the specific staining of keratinocytes in normal skin (BioChain, Catalog No: T2234218, Lot. C105169) (Fig. 2c). Isotype control antibodies showed no staining in all samples used for IHC in this report (data not shown). To further confirm the expression of IL-34 in these samples, total RNAs were collected from formalin-fixed paraffin-embedded (FFPE) samples (NucleoSpin total RNA FFPE XS, Macherey-Nagel, Catalog No: 740969.10) and then reverse-transcribed into cDNA (ReverTraAce qPCR RT Master Mix, Toyobo, Catalog No: F0937K). RT-PCR analysis on these samples showed elevated levels of IL-34 mRNA in addition to several inflammatory cytokines such as interleukin (IL)-1β, IL-6, IL-8, and tumor necrosis factor α (TNFα) in the inflammatory cyst of the submandibular gland compared to normal salivary gland tissues (Additional file 1: Figure S1).

**Fig. 3:**
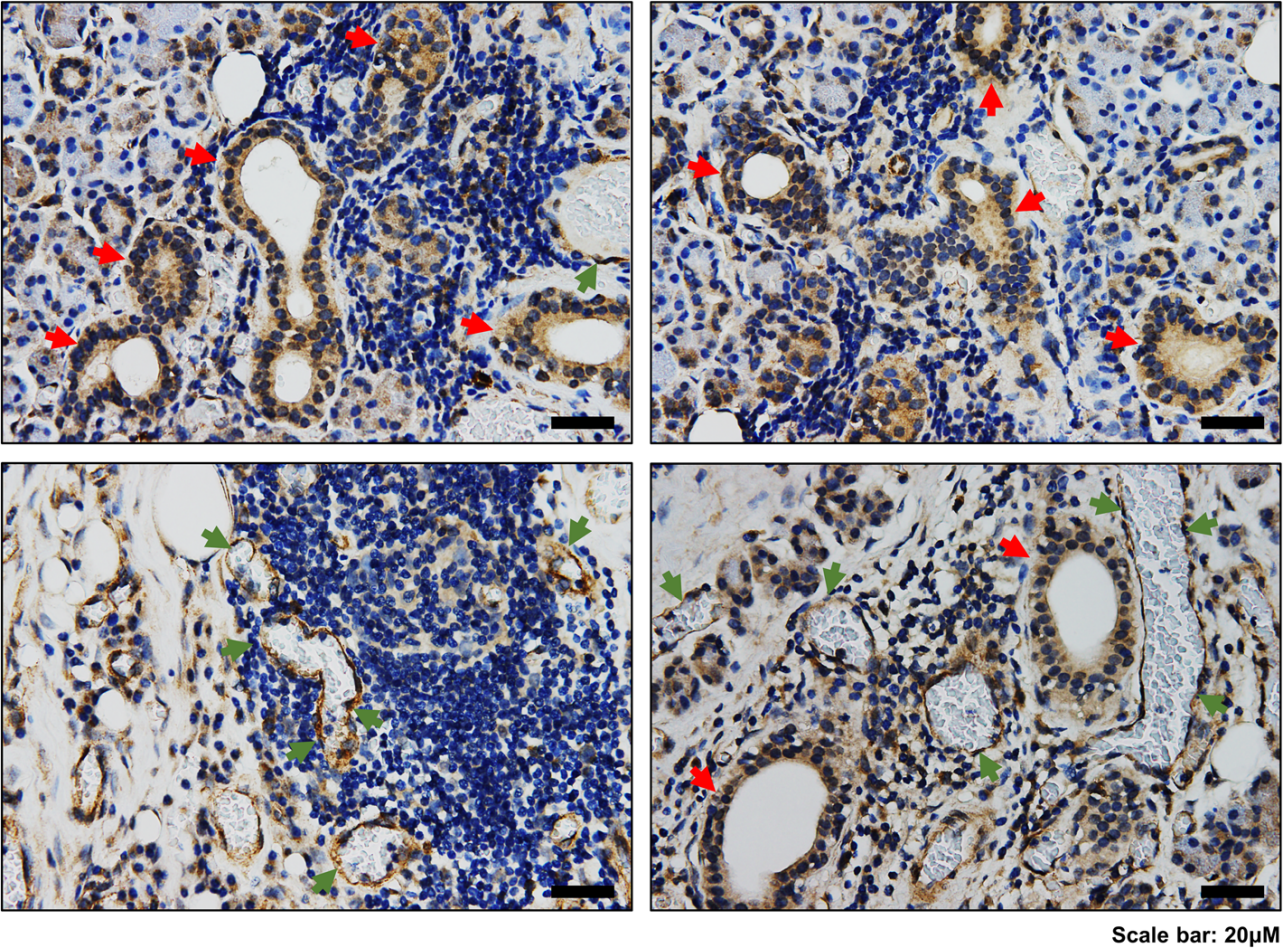
High expression of IL-34 in ductal epithelial cells and endothelial cells of blood vessels correlates with high infiltration of immune cells. Representative data of IL-34 staining by IHC showing the localized expression of IL-34 in ductal epithelial cells and endothelial cells of blood vessels. Hematoxylin staining demonstrates lymphocyte accumulation around IL-34-expressing epithelial (red arrows) or endothelial (green arrows) cells. Scale bars, 20 μM

Consistent with this inflammatory condition, high expression of IL-34 in the affected gland showed a tendency to be accompanied with high infiltration of lymphocytes, as confirmed in both cases of the epithelium of the ductal system as well as endothelium of blood vessels (Fig. 3). Collectively, these results indicate a potential involvement of IL-34 in the inflammatory response observed in this case of the inflamed submandibular gland.

## Discussion

In this case report, we introduce for the first time a case of inflammatory cyst within the submandibular gland that showed enhanced expression of IL-34 accompanied by high infiltration of lymphocytes. In normal tissues of salivary glands, IL-34 showed very weak expression in the ductal epithelium as confirmed by IHC. This is consistent with the findings of a recent study that described the existence of IL-34 in normal saliva at very low concentrations [10, 11]. The expression of IL-34 was significantly increased in tissues surrounding the inflammatory cyst of the inflamed submandibular gland and specifically localized in ductal epithelial cells in addition to endothelial cells of blood vessels. Remarkably, high expression of IL-34 in epithelial and endothelial cells showed a tendency to be accompanied by increased immune infiltration.

Although IL-34 expression was first described in neurons and keratinocytes, several studies have reported the expression of IL-34 in a wide range of cells, which seems to be pathological rather than physiological, such as in immune cells, synovial fibroblasts, adipocytes, and cancer cells [6]. This is supported by in vitro evidence, where IL-34 expression can be induced or enhanced upon cellular stress, such as in cancer cells treated with chemotherapeutic agents, fibroblasts stimulated with pro-inflammatory cytokines such as TNFα and IL-1β, and hepatocytes infected with HCV [6]. In most cases, the enhancement of IL-34 expression is regulated by NF-κB, a central molecule that controls inflammatory signaling pathways [6]. Thus, the enhancement of IL-34 expression showed in this case report is consistent with the correlation between IL-34 and inflammation.

A remarkable observation in this case report is the high expression of IL-34 in endothelial cells. An angiogenic function of IL-34 was described in a murine model of osteosarcoma. Osteosarcoma cell-derived IL-34 promoted new vessel formation and extravasation of immune cells. Interestingly, stimulation of human endothelial cells such as ECFCs and HUVECs with recombinant IL-34 activates ERK1/2 and FAK signaling pathways, resulting in enhanced proliferation and tubular morphogenesis. Furthermore, IL-34 increased the adherence of immune cells to activated endothelium under shear stress conditions [12]. By reflecting these backgrounds on the observations described in this case report, endothelial cell-derived IL-34 is expected to have a dual function during the inflammatory process. First, IL-34 acts in an autocrine-dependent manner by increasing proliferation of endothelial cells and thus promoting the formation of new blood vessels. In a paracrine-dependent manner, IL-34 secondly increases adherence of immune cells to endothelial cells and thus accelerates extravasation and accumulation of immune cells into the inflammation foci.

Several studies on the physiological and pathological functions of IL-34 are gradually establishing the correlation between IL-34 and inflammation in its two phases, acute or chronic [6]. Pro-inflammatory cytokines can induce the expression of IL-34 in several cells, which in turns can induce the expression of other inflammatory cytokines [6]. Together with its angiogenic role, IL-34 can be expected to play central roles in the amplification of the inflammatory cycle.

Growing evidence from basic and clinical studies has indicated the involvement of IL-34 in tumors in all aspects of the tumor microenvironment, including tumor growth, invasion, angiogenesis, metastasis, immunosuppression, and therapeutic resistance [6]. In this regard, it is of great interest to examine the expression of IL-34 in tumors of the salivary glands such as pleomorphic adenoma and adenoid cystic carcinoma and evaluate the possible involvement of IL-34 in the pathogenicity of these diseases in future works.

## Conclusion

In conclusion, the present study provides evidence for the first time of enhanced IL-34 expression in ductal epithelial cells and endothelial cells accompanied with increased immune infiltration within the inflammatory foci of the inflamed salivary gland. Future works are required to confirm the pathogenic role of IL-34 in modulating the immune inflammatory pathways in salivary gland disorders.

## Additional file

Additional file 1: **Figure S1.** Enhanced expression of IL-34 and several inflammatory cytokines in the inflammatory cyst of the submandibular gland. RT-PCR analysis shows elevated levels of IL-34 mRNA, which accompanies the enhancement of inflammatory cytokines expression such as IL-1β, IL-6, IL-8, and TNFα in the inflammatory cyst of the submandibular gland compared to normal salivary gland tissues (TIF 2149 kb)

## Abbreviations

CSF-1R: Colony stimulating factor-1 receptor
ECFCs: Endothelial Colony Forming Cells
ERK: Extracellular signal-Regulated Kinase
FAK: Focal Adhesion Kinase
FFPE: Formalin-fixed paraffin-embedded
H & E: Hematoxylin and eosin staining
HCV: Hepatitis C virus
HUVECs: Human Umbilical Vein Endothelial cells
IHC: Immunohistochemistry
IL-1β: Interleukin-1β
IL-34: Interleukin-34
MRI: Magnetic resonance imaging
NF-κB: Nuclear factor-Kappa Beta
RT-PCR: Reverse transcription polymerase chain reaction
TNFα: Tumor necrosis factor α
