# Supplementary figures and images for: Enhanced expression of IL-34 in an inflammatory cyst of the submandibular gland: a case report
Source: Inflamm Regen. 2018 Jul 10;38:12. doi: 10.1186/s41232-018-0069-6 (PMC6038191; doi:10.1186/s41232-018-0069-6)

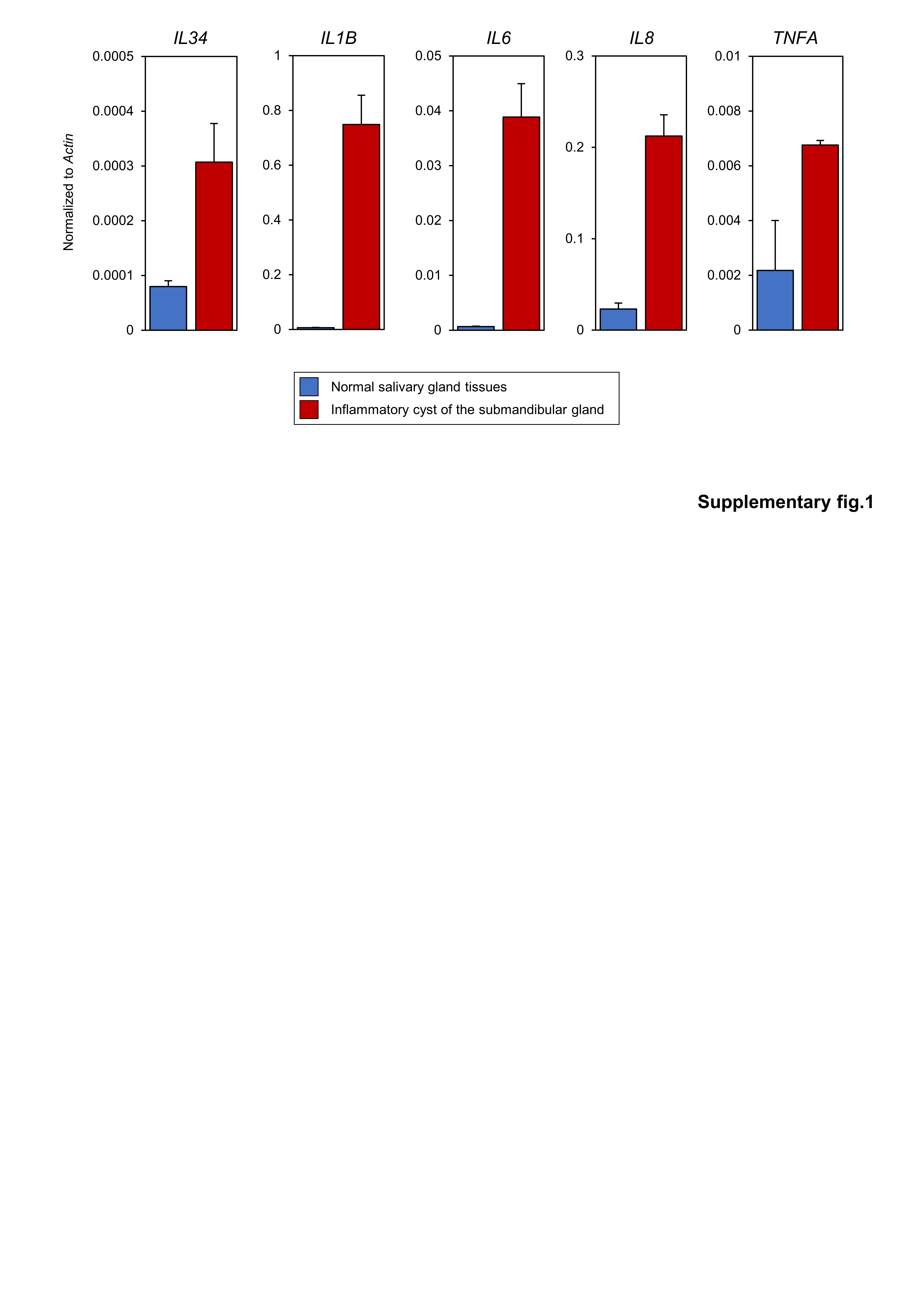

Supplement: Supplementary file 1 — Figure S1. Enhanced expression of IL-34 and several inflammatory cytokines in the inflammatory cyst of the submandibular gland. RT-PCR analysis shows elevated levels of IL-34 mRNA, which accompanies the enhancement of inflammatory cytokines expression such as IL-1β, IL-6, IL-8, and TNFα in the inflammatory cyst of the submandibular gland compared to normal salivary gland tissues (TIF 2149 kb) [file 41232_2018_69_MOESM1_ESM.tif]
